# Exploring Performance Calibration in Relation to Better or Worse Than Average Effect in Physical Education

**DOI:** 10.5964/ejop.v14i3.1599

**Published:** 2018-08-31

**Authors:** Athanasios Kolovelonis, Eleni Dimitriou

**Affiliations:** a Department of Physical Education and Sport Science, University of Thessaly, Trikala, Greece; Department of Psychology, Webster University Geneva, Geneva, Switzerland; Institute of Psychology, University of Wroclaw, Wroclaw, Poland

**Keywords:** calibration bias and accuracy, better or worse than average effect, physical education, performance estimations

## Abstract

The aim of this study was to explore students’ calibration of sport performance in relation to better or worse than average effect in physical education settings. Participants were 147 fifth and sixth grade students (71 boys, 76 girls) who were tested in a soccer passing accuracy test after they had provided estimations for their own and their peers’ performance in this test. Based on students’ actual and estimated performance, calibration indexes of accuracy and bias were calculated. Moreover, students were classified in better, worse, or equal than average groups based on estimated scores of their own and their peers’ average performance. Results showed that students overestimated their own performance while most of them believed that their own performance was worse than their peers’ average performance. No significant differences in calibration accuracy of soccer passing were found between better, worse, or equal than average groups of students. These results were discussed with reference to previous calibration research evidence and theoretical and practical implications for self-regulated learning and performance calibration in physical education.

Over the past few decades, research in various domains has been interested in examining how people judge their knowledge, ability, or performance in various types of tasks. The roots of this research have been traced in decision making literature examining probability judgments and accuracy (e.g., Keren, 1991) as well as in the domain of metacognitive research focusing in students’ awareness of their learning or performance (e.g., Hacker, Bol, & Keener, 2008). Research on these domains has focused on exploring the underlying mechanisms involved in metacognitive monitoring and the factors contributing in the discrepancy between estimated and actual performance. Such research regarding aspects of metacognition including the accuracy of performance estimations has also recently emerged in sport (e.g., Fogarty & Else, 2005) and physical education (e.g., Kolovelonis & Goudas, 2018) settings. The present study followed and expanded this trend focusing on the interactions between students’ estimation of their own and their peers’ performance.

One consistent finding in this line of research is people’s tendency to overestimate their knowledge, ability, or performance (e.g., Chen, 2003). According to Moore and Healy (2008) this overconfidence can take three distinct forms, namely overestimation, overplacement, or overprecision. In particular, overestimation is people’s tendency to estimate their own performance higher than it actual is. The typical research paradigm for examining this aspect of overconfidence is to ask students to provide an estimation for their performance in a test and then to compare this estimation with their actual performance. The discrepancy between estimated and actual performance, also called calibration, can take the direction of overestimation or underestimation of performance. Overplacement refers to students’ belief that they are better than the majority of their peers. The term better or worse than average is also used to capture students’ beliefs regarding their own and their peers’ performance estimating that they are better or worse than average. Overprecision refers to students’ certainty regarding the accuracy of their beliefs about their answers. The questions asked have usually numerical answers and students should estimate the correct answers using 90% confidence intervals. The present study focused on the calibration of self and peers’ performance in sport tasks in physical education. In particular, associations between the accuracy of estimations of self performance (i.e., calibration) and the estimation of the peers’ performance taking the form of better or worse than average effect were examined.

## Calibration of Performance: Conceptualization and Research Evidence

Calibration is the degree to which a person’s perception of performance corresponds with his or her actual performance (Keren, 1991). The comparison between estimated and actual performance can produce accuracy or inaccuracy in two different directions: overestimation or underestimation. The way students estimate their performance may affect their motivation to be involved in certain activities and their efforts to self-regulate their learning (Efklides & Misailidi, 2010). For example, a student who erroneously estimates that his performance is high may decrease his learning efforts, whereas a student who underestimates his capabilities may avoid challenging tasks. In both cases, students may limit their potential for enhancing their learning and performance.

There is a long tradition of calibration research in academic settings. This research has shown that students are often inaccurate when judge their performance (e.g., Chen, 2003; Hacker & Bol, 2004) and this miscalibration usually has the direction of overconfidence (e.g., Eme, Puustinen, & Coutelet, 2006; Hacker, Bol, & Bahbahani, 2008). Moreover, calibration accuracy is related with higher levels of performance (e.g., Bol, Hacker, O’Shea, & Allen, 2005; Ots, 2013) and can be improved with feedback (e.g., Labuhn, Zimmerman, & Hasselhorn, 2010) and practice (e.g., Bol, Hacker, Walck, & Nunnery, 2012).

In sport settings, only a few studies have examined performance calibration. Golfers were well calibrated on easier tasks (putting) but overconfident on more difficult tasks (chipping and pitching) (Fogarty & Else, 2005), and recreational basketball players overestimated their shooting performance (McGraw, Mellers, & Ritov, 2004). In physical education, students were generally overconfident regarding their basketball dribbling performance (Kolovelonis, Goudas, & Dermitzaki, 2012), and performance calibration did not differ between students who practiced dribbling receiving social feedback and setting process or performance goals and control group students (Kolovelonis, Goudas, Dermitzaki, & Kitsantas, 2013). Recent research in physical education has shown that person related factors, such as task orientation and self-efficacy (Kolovelonis & Goudas, 2018) were associated with students’ capability to estimate accurately their performance.

## Estimating Peers’ Performance: Am I Better or Worse Than Average?

Except of judgments regarding the self, social comparative judgments have also attracted researchers who are interested in examining the accuracy and bias of peoples’ judgments (Chambers & Windschitl, 2004). It has been suggested that group settings and social comparisons can influence metacognitive judgments (Bol & Hacker, 2012). The term “better or worse than average” has been used to describe students’ tendency to believe that their performance is above or below average performance (Kruger & Dunning, 1999). Research has shown that people have usually positive views when evaluate themselves compared to others. For example, a review of studies regarding students’ self-assessments of skill and character in three domains (i.e., health, education, and workplace) concluded that people tend to believe that they are above average (Dunning, Heath, & Suls, 2004). It has been proposed that this tendency can be explained by people’s desire to protect, enhance, or restore feelings of self-worth (e.g., Brown, 2012). However, variations in this tendency can also be found. Evidence has suggested that people may rate themselves below average in some domains in which success is rare (e.g., Kruger, 1999). For example, in difficult tasks, people may tend to overestimate their performance believing that they are worse than others, while in easy tasks people usually underestimate their performance believing they are better than others (Moore & Healy, 2008). Thus, except of motivational factors (e.g., self-enhancement), cognitive mechanism may also be involved in producing the better or worse than average effect (e.g., Chambers, 2008; Moore & Small, 2007).

Furthermore, students’ mindset, that is their beliefs regarding the fixedness or malleability of their personal competences (Dweck & Molden, 2005), play a key role in their motivation and achievement and it may be involved in calibration of their own or their peers’ performance. In particular, students who believe that their competences or intelligence can be developed (i.e., a growth mindset) tend to outperform those who believe that their competences or intelligence are fixed (i.e., a fixed mindset) (Dweck, 2017). Interventions focusing on promoting a growth mindset were beneficial for poorly performing students (Paunesku et al., 2015). Moreover, it was found that a growth mindset was associated with improved performance and decreased calibration error (Ravenscroft, Waymire, & West, 2012). Thus, promoting a growth mindset may help students to improve performance and decrease calibration error (Dweck, 2017) contributing to the reversal of the underachievement among students (Wong, 2016).

Educational environments such as the physical education settings are contexts that students judge not only their own performance but also the performance of their classmates (Labuhn et al., 2010). In fact, peers become increasingly important as students grow up (Schunk & Meece, 2006) and can function as models affecting their motivation and behavior. For example, a student who observes their classmates succeed in a new task may feel more self-efficacious and motivated to try this task (Schunk & Pajares, 2009). That is, students tend to compare their performance with the performance of their classmates and these judgments may be used by students for building their own perceptions of ability (Dunning & Hayes, 1996). Indeed, self-perceptions of competence involve social comparison (i.e., in what position I rank myself compared to others or how other people perceive my ability) (Dermitzaki & Efklides, 2000). These social comparisons are inevitable and occur even if students are not explicitly asked for doing such comparisons. For example, in a physical education class students are likely to compare their performance with the performance of their classmates to draw conclusions about how well they performed the sport task at hand. These social comparisons are facilitated by the nature of the sport tasks where students’ knowledge, skill, and performance are apparent to all participants within a class. Therefore, judgments regarding the performance or the competence of others can be considered to have a metacognitive nature (Efklides, 2011), may be associated with judgments regarding self performance and may also be subject to bias. That is, a student may inaccurately believe that their classmates are better than he is in a sport task. Thus, considering the important implications of social comparisons in forming metacognitive judgments, the examination of the accuracy of students’ estimations regarding their peers’ performance is of great interest.

## The Present Study

Although research following the calibration paradigm in sport and physical education has recently increased (e.g., Fogarty & Else, 2005; Kolovelonis & Goudas, 2018), further research is needed to explore calibration in relation to other metacogntive judgments of performance. Moreover, research regarding the associations of social comparisons and the accuracy of students’ metacognitive judgments is warranted (Bol & Hacker, 2012). Thus, this study focused on the relations between students’ calibration of their own performance and their beliefs about the performance of their classmates (i.e., better or worse than average effect). Such associations are still unexplored in sport and physical education settings and may inform the research regarding the construct of calibration of performance and the factors associated with it including social comparisons processes.

Some research in academic settings has shown associations between better or worse than average effect and performance calibration. Larrick, Burson, and Soll (2007) found a positive relationship between better or worse than average effect and overconfidence among university students who responded questions of general knowledge. Higher perceptions of ability relative to others predicted greater degrees of overconfidence. Moreover, students who believed that their actual performance was higher than their peers’ performance (i.e., in terms of higher percentile) were more overconfident. Burson, Larrick, and Klayman (2006) examining participants’ judgments of performance relative to their peers found that both skilled and unskilled performers (defined by performance on the tasks) were inaccurate in their estimations of performance in a trivia quiz. Furthermore, evidence has suggested than the manipulation of social comparisons (even regarding the performance of a fictitious group of classmates) affected metacognitive judgments with greater magnitude in judgments associated with higher performance (De Carvalho Filho & Yuzawa, 2001).

Students’ beliefs regarding peers’ performance may have implications for their involvement in physical education. For example, students may perform less than they actually can in order to avoid becoming socially isolated in the case they may be perceived by their peers as overly intelligent (Schunk & Pajares, 2009). Beliefs you are better or worse than average may also be involved in the decision to participate in an activity. Moreover, considering that students may use the competence of others for forming their own estimations of performance, miscalibration of their peers’ performance may be related with miscalibration of their own performance. Experimental manipulations focusing on group interactions and social comparisons (i.e., practicing in group settings and guidelines) were effective in promoting calibration accuracy and achievement (Bol et al., 2012). Thus, calibration research should focus not only on how students estimate their own performance but also on how accurate their estimations for their peers’ performance are.

Moreover, the way students interpret information regarding peers’ performance and the potential effects of these perceptions on self-regulated learning is an important research question that warrants further exploration (Labuhn et al., 2010). For example, it has been found that normative feedback informing that one’s own performance is below average may decrease self-efficacy and task interest (e.g., Kavussanu & Roberts, 1996) while positive comparisons with the ‘norm’ can enhance self-efficacy, produce positive self-reactions, and increase motivation to practice a skill (e.g., Hutchinson, Sherman, Martinovic, & Tenenbaum, 2008). Manipulating participants’ beliefs, Wulf, Shea, and Lewthwaite (2010) found that participants who were led to believe that their performance was better than average, demonstrated more effective learning than participants who were led to believe that their performance was worse than average. That is, students’ learning and performance may be influenced by the perception of being better or worse than their classmates representing a self-fulfilling prophecy. Therefore, examining associations between performance calibration and the better or worse than average effect can further shed light in the dynamics of metacognitive estimates in students’ learning and performance.

The aim of this study was to examine the associations between students’ calibration bias and accuracy of sport performance in relation to their beliefs that they were better or worse than the average performance of their classmates. Due to the exploratory nature of this study in the domain of physical education non specific hypotheses were stated.

## Method

### Participants

Participants were 147 Greek students (*M*_age_ = 11.28, *SD* = 0.65, 71 boys, 76 girls) who attended three fifth grade (64 students, 33 boys, 31 girls) and four sixth grade (83 students, 38 boys, 45 girls) physical education classes from three elementary schools located in two middle-sized cities in central Greece.

### Measures

#### Soccer Passing Test

A modified soccer passing accuracy test was used (Ali, 2011). Students, from a 10 meter distance, executed without time limit 5 passes trying to pass the ball through two cones one meter apart. The number of the successful passes was each student’s score in this test.

#### Estimations of Soccer Passing Performance

Before performing the test, standing in the passing position, students were asked to estimate their own and their peers performance in the passing test responding the following questions: “How many passes out of 5 you will pass though the cones from this position?” and “How many passes out of 5 your classmates on average will pass though the cones from this position?” Students’ answers in these two questions were their scores in estimation of their own and their peers’ performance in the soccer test, respectively.

#### Calibration Indexes

Indexes of calibration bias and accuracy for students’ performance were calculated. In particular, calibration bias for passing performance was computed as students’ estimated minus actual performance in the passing test. Calibration bias is an index of the direction of the calibration with positive scores indicating overestimation of performance and negative underestimation. Based on their scores in the bias index students were classified in three groups (i.e., accurates, overestimators, and underestimators). The absolute values of the bias scores resulted in the accuracy index which reflects the magnitude of calibration error. Values closer to zero in the accuracy index indicate higher calibration accuracy (Schraw, 2009).

#### Better or Worse Than Average Index

An index representing the better or worse than average effect was computed for each student as students’ estimation of their own performance minus their estimation regarding peers’ average performance. Students with positive scores in this index were classified in the better than average group while students with negative scores were classified in the worse than average group. Students with score zero were classified in the equal group. This index represents students’ beliefs regarding their own performance with respect to their perceptions regarding peers’ average performance.

### Procedures

Ethical approval for this study was granted by the University Ethics Review Committee. Moreover, school principals and physical education teachers provided their permission and parental consents were obtained. Students participated in the study voluntarily at individual level. The experiment took place during regular physical education lesson and students were informed that they would perform a soccer passing test consisted of 5 passes. Next, students standing in the passing position were asked to estimate their own performance (i.e., number of successful passes) and the performance of their peers (i.e., number of successful passes of their classmates on average). Then, students were provided with oral instructions and observed the experimenter’s passing demonstration, performed trial passes for a minute, and were tested in passing.

### Statistical Analyses

The accuracy index was used as dependent variable in the analyses of variance for comparing groups while the bias scores were used for classifying students as accurates (score: zero), underestimators (negative scores), and overestimators (positive scores) (Gonida & Leondari, 2011). Regression analyses were conducted to examine if students scores in better or worse than average index could predict their scores in calibration bias and calibration accuracy indexes. Analyses of variance were conducted for comparing better, equal, or worse than average groups in calibration accuracy. Crosstabulations analysis and chi square tests were used for comparing frequencies of acurates, underestimators, and overestimators and better, worse, or equal than average groups. Effect sizes of η^2^ and Cohen’s *d* were also calculated (Cohen, 1988).

## Results

### Preliminary Analyses

Means and standards deviations of all variables are presented in Table 1. No differences between genders, *t*(145) = -0.60, *p* = .552, or grades, *t*(145) = -0.77, *p* = .441, in calibration accuracy in soccer passing were found. Therefore, gender and grade was not considering as a factor in the rest analyses. Regarding calibration bias, overestimators (*n* = 60) were more compared to accurates (*n* = 46) and underestimators (*n* = 41), but nonsignificant difference in these frequencies was found, χ^2^(2, *N* = 147) = 3.96, *p* = .136.

### Correlations and Regressions

Intercorrelations of the study variables are presented in Table 1. Students’ estimations for their own and their peers’ performance were not correlated.

Regression analyses showed that students’ scores in better or worse than average index could significantly predict their scores in calibration bias, *R* = .44, *F*(1, 145) = 34.38, *p* < .001, and calibration accuracy, *R* = .18, *F*(1, 145) = 4.60, *p* = .034.

**Table 1:** Means, Standards Deviations, and Correlations Between Study Variables

Variable	Correlations						
	*M*	*SD*	1	2	3	4	5
1. Soccer passing performance	2.33	1.33	-				
2. Estimation of self performance	2.67	1.21	.32**	-			
3. Estimation of peer performance	3.44	0.94	.12	.16	-		
4. Self-calibration bias	0.35	1.48	-.64**	.53**	.03	-	
5. Self-calibration accuracy	1.14	1.01	-.18*	.26**	.07	.37**	
6. Better or worse than average index	-0.77	1.40	.20*	.75**	-.53**	.44**	.18*

*p < .05. **p < .01.

### Comparisons Between Better, Equal or Worse Than Average Groups

Frequencies of better, equal or worse than average groups were calculated and chi square showed significant differences between groups, χ^2^(2, *N* = 147) = 70.69, *p* < .001. The majority of students were classified in the worse than average group (*n* = 97) and the rest in the better than average (*n* = 23) or in the equal than others (*n* = 27). Moreover, crosstabulations analysis of frequencies of better, worse, or equal than average groups and calibration bias groups (accurates, underestimators, and overestimators) showed significant differences, χ^2^(4, *N* = 147) = 14.88, *p* = .005. In particular, within the better and equal than average groups, students who overestimated their performance were more than accurates and underestimators (Table 2).

No differences in calibration accuracy of soccer passing, *F*(2, 144) = 2.56, *p* = .081, were found between groups of students classified as better (*M* = 1.57, *SD* = 1.20), worse (*M* = 1.07, *SD* = 0.94), or equal (*M* = 1.00, *SD* = 1.04) than average.

**Table 2:** Crosstabulations of Frequencies of Better or Worse Than Average Groups and Bias Groups

Self-bias groups	Better or worse than average groups			Total
	Equal	Worse	Better	
Accurates	9	31	6	46
Underestimators	5	35	1	41
Overestimators	13	31	16	60
Total	27	97	23	147

## Discussion

A growing interest for calibration research in the domain of physical education has recently emerged (e.g., Kolovelonis & Goudas, 2018). The present study, following and expanding this trend, focused not only in the accuracy of students’ estimations of their own performance but also on students’ estimations regarding the performance of their classmates. Both of these types of metacognitive estimations are matter because they are involved in the process of students learning and performance in physical education. Next, the results of the present study are discussed with reference to previous evidence in calibration research and theoretical and practical implications for learning and performance in physical education.

### Accuracy in Estimating Self and Peer Performance

Students overestimated their own performance (i.e., estimated performance was 15% higher than the actual) and overestimators were more compared to accurates and underestimators. These results are consistent with previous evidence showing an overconfidence effect when students estimate their performance in academic (Chen, 2003; Hacker & Bol, 2004; Hacker, Bol, & Bahbahani, 2008), sport (Fogarty & Else, 2005; McGraw et al., 2004), and physical education (Kolovelonis & Goudas, 2018; Kolovelonis, Goudas, & Dermitzaki, 2012) settings. Moreover, students overestimated their peers’ average performance. In fact, in the total sample students estimated that their peers’ average performance would be 48% higher than the actual mean performance of the participants in this study was. These results showed that students tended to overestimate not only their own performance but also the performance of their peers supporting views that people usually have imperfect knowledge of both their own and their peer’ performance (Moore & Healy, 2008). Moreover, this inaccuracy in estimating self and peers’ performance may imply that these two forms of metacognitive judgments (i.e., estimations of self and peer performance) interact and maybe based on common underlying metacongitive processes (Efklides, 2011). The lack of metacognitive self-awareness may be related not only with difficulties in estimating self performance but also may be involved in social comparisons process. These interpretations are also discussed below in relation to better or worse than average effect and should be further explored in future research.

### Calibration of Performance and the Better or Worse Than Average Effect

This study focused on students’ performance calibration in relation to the better or worse than average effect. The majority of students (almost two thirds of students) reported that their peers’ performance would be higher compared to their own performance and thus they were classified in the worse than average group. This result was not consistent with previous research showing that people tend to believe that their abilities in various domains (i.e., health, education, and workplace) were above average (Dunning et al., 2004). However, other views have also suggested that oftentimes people claim that they are worse than others (Chambers, 2008) especially when they judge their ability for difficult tasks, like computer programming (Kruger, 1999) or difficult sport tasks (Vanyperen, 1992). Similarly, it has been suggested that on difficult tasks, people usually believe that they are worse than others while on easy tasks better than others (Moore & Healy, 2008) indicating that the difficulty of the task may affect the direction of the better or worse that average effect. Considering this evidence, a possible explanation of the results of this study may be associated with the nature of the sport task used. In particular, in the case of sport tasks immediate and apparent feedback regarding performance is available not only to performers but also to observers. This made the process of social comparison easier for most students and helped them to make conclusions regarding the difficulty of the task. Students in the present study may consider the soccer passing task as a difficult one and thus they believed that their performance would be lower compared to their peers’ performance. Other explanations have been also proposed for the better or worse than average effect including both motivational (e.g., self-enhancement, self-protect) and cognitive factors (e.g., egocentrism, selective accessibility in information about the self and others) (Chambers, 2008). For example, some students may have inflated their estimations for peers’ performance to protect their self-worth from unfavorable social comparative judgments in the case of their performance was low. However, such interpretation should be experimentally examined in future research exploring further the nature of better or worse than average effect and the factors associated with it in physical education.

An interesting result was that students who were classified in the better than average group were less accurate in estimating their own performance compared to students who classified in the worse, or in equal than average groups. However, this difference did not reach significance. Moreover, two third of students who classified in the better than average group overestimated their own performance while students’ scores in the better or worse than average index could significantly predict their scores in calibration bias and accuracy. All these results in combination suggest that the way students perceived and estimated their performance in relation to their peers’ performance may be related with the calibration accuracy of their own performance. Previous research has shown that students who overestimated their own performance compared to their peers’ performance were more overconfident (Larrick et al., 2007). Indeed, it has been proposed that performance calibration may affected by social factors (Schunk & Pajares, 2004). That is, students may use social comparison to form estimations for their own performance. Thus, if one’s estimations of peers’ performance are inaccurate he or she may also estimate inaccurately their own performance. However, considering the cross-sectional nature of this study, no cause and effect relations could be established by the present results. Further research is needed to explore the dynamic and interactive relationships between metacognitive judgments of self and peers’ performance.

### Calibration, Better or Worse Than Average Effect, and Self-Regulated Learning

The results of this study may inform processes involved in students’ self-regulated learning development. During the first phases of self-regulated learning development students are based on social support (i.e., modeling, social feedback) to develop their skills (Goudas, Kolovelonis, & Dermitzaki, 2013; Zimmerman, 2000). Thus, the way students interpret information from social environment may be crucial for the development of self-regulated learning (Labuhn et al., 2010). In fact, comparisons with the ‘norm’ can produce positive or negative self-reactions and affect self-efficacy and motivation to practice a skill (e.g., Hutchinson et al., 2008). In the present study almost all students had a misleading picture for the “norm” as miscalibrated their peers’ performance (i.e., they estimated peers’ performance higher than it actually was) believing that their performance would be lower than their peers’ performance. Thus, the accuracy of students’ estimations of their classmates’ performance is crucial in the sense that these estimations are the basis in the social comparison process and can affect students’ perceptions of their own abilities (Dunning & Hayes, 1996). For example, miscalibration of peers’ performance may affect students’ beliefs regarding their own abilities (Kavussanu & Roberts, 1996) and their motivation to be involved in self-regulated practice (Zimmerman, 2000).

Moving towards mastering the skill students rely more on producing self-feedback through self-monitoring processes (Goudas et al., 2013; Zimmerman, 2000). In fact, research in physical education has shown that self-monitoring process in the form of self-recording of performance had a positive effect on students’ performance (Kolovelonis, Goudas, & Dermitzaki, 2011; Kolovelonis, Goudas, Hassandra, & Dermitzaki, 2012). However, the accuracy of these metacognitive judgments (i.e., self-monitoring) regarding performance is crucial for the development of self-regulated learning. Indeed, previous findings in physical education has shown that the accuracy of feedback students receive through the practice with the reciprocal and the self-check teaching styles was positively associated with their performance (Kolovelonis & Goudas, 2012). However, the majority of students in this study miscalibarated their performance, either being overestimators or underestimators. This miscalibration may affect students’ motivation to be involved in practicing and their efforts to self-regulate their learning (Efklides & Misailidi, 2010). Thus, improving students’ calibration, by implementing interventions such as those describing in the next section, should help them to accurately estimate their performance and to enhance their self-regulated learning and performance.

### Practical Implications

The results of this study can inform practical implications for physical education. Teachers should help students to be more accurate in estimating both their own and their peers’ performance designing interventions for calibrating these estimations. Such interventions should focus on increasing students’ awareness regarding their own and their peers’ performance reducing thus the discrepancy between estimated and actual performance. In fact, practicing calibration (Bol et al., 2012) and providing students with feedback (Labuhn et al., 2010) have been found to improve students’ capability to estimate their performance accurately. Moreover, focusing on personal improvement (i.e., promoting students’ task orientation) rather than on trying to outperform others may help students to be better calibrated. In fact, a recent study in physical education showed that task orientation was a significant predictor of students’ calibration accuracy (Kolovelonis & Goudas, 2018). Furthermore, involving students in self-regulatory practice (Kolovelonis, Goudas, & Dermitzaki, 2010) including metacognitive monitoring and self-recording (Kolovelonis, Goudas, & Dermitzaki, 2011) may help them to increase their awareness regarding their performance and to become well-calibrated students. The use of teaching styles, such as the reciprocal and the self-check styles (Mosston & Ashworth, 2002), can also help students to improve performance and to increase their awareness regarding their performance and the standards to judge their own and their peers’ performance in sport tasks (Kolovelonis, Goudas, & Gerodimos, 2011). Moreover, considering that changing students’ mindsets can enhanced their achievement and decreased their calibration error (Dweck, 2017; Ravenscroft et al., 2012) students should be taught that personal competencies and intelligence are malleable rather than fixed.

### Limitations and Future Research

This study was cross-sectional in nature and thus no cause and effects relationships can be established from the present results. Future research should involve longitudinal designs to explore the dynamic interactions between students’ estimations of their own and their peers’ performance. Previous research has shown that the relationships between calibration of performance and the better or worse than average effect may be vary based on the task difficulty (Moore & Healy, 2008). Thus, future research should consider the potential effects of the difficulty of the tasks in the association between calibration and better or worse than average effect. Such research should also involve students’ perceptions of the task difficulty as the feelings of difficulty have been negatively associated with basketball shooting performance in physical education (Goudas, Dermitzaki, & Kolovelonis, 2017). In this study, students’ classification in better, worse, or equal than average groups was based on the estimation of their peers’ performance which is considered an indirect method of calculating this index (Chambers & Windschitl, 2004). Future research should also use direct methods of measuring the better or worse than average effect (e.g., asking students if they consider themselves better or worse than average in a specific task) (e.g., Hamamura, Heine, & Takemoto, 2007). Although both methods have been successfully used in documenting the better than average effect, the direct method seems to yield stronger evidence than the indirect (Chambers & Windschitl, 2004). Associations between calibration, the better or worse than average effect, and students’ learning and performance in motor and sport tasks should also be examined. For example, interventions enhancing students’ beliefs that their performance was better than average had positive effects on students’ learning and performance (Wulf et al., 2010). This result implies that students’ self-efficacy beliefs may be involved in the process of calibrating their performance. Indeed, a recent study showed that self-efficacy predicted students’ calibration accuracy in physical education (Kolovelonis & Goudas, 2018). Thus, potential associations between better or worse than average effect, students’ calibration, and their self-efficacy beliefs should be further explored in future research.
